# A Unified Deep-Domain Adaptation Framework: Advancing Feature Separability and Local Alignment

**DOI:** 10.3390/s25123671

**Published:** 2025-06-12

**Authors:** Pranav Kumar, Jimson Mathew, Rakesh Kumar Sanodiya, Avinash Kumar Chouhan, Rahul Reddy Bukkasamudram, Chandra Sai Teja Adhikarla

**Affiliations:** 1 Department of Computer Science and Engineering, Indian Institute of Technology Patna, Bihar 801106, India; jimson@iitp.ac.in; 2 Department of Computer Science and Engineering, Indian Institute of Information Technology, Design and Manufacturing, Jabalpur 482005, India; 3 North Eastern Space Applications Centre Umiam, Meghalaya 793103, India; avinash.chouhan@nesac.gov.in; 4 Department of Computer Science and Engineering, Indian Institute of Information Technology, Sri City, Chittoor, Sri City 517646, India; rahulreddy.b21@iiits.in (R.R.B.); chandra.a21@iiits.in (C.S.T.A.)

**Keywords:** transfer learning, domain adaptation, image classification, attention mechanism, entropy loss

## Abstract

In transfer learning, domain adaptation is one of the key research areas. For domain adaptation, domain shift is a known problem when the data distribution of the source domain, from which the training data is fetched, and the target domain, from which the test data is fetched, vary significantly. Aligning the source and target domains is a solution, but due to alignment, the intrinsic properties of the data may be altered. To address this issue of domain shift, we introduce a novel method, called “A Unified Deep-Domain Adaptation Framework: Advancing Feature Separability and Local Alignment” (DDASLA) that incorporates an attention mechanism into the ResNet18 model to improve its feature extraction capability. Apart from self-attention, a combined loss function consisting of angular loss, Local Maximum Mean Discrepancy (LMMD), and entropy minimization is used. Angular loss enhances feature discrimination through angular alignment, whereas LMMD equalizes local data distributions across domains, and entropy minimization refines the decision boundaries. A comprehensive experiment on the Office and remote sensing datasets shows that DDASLA outperforms several state-of-the-art methods. These findings show that DDASLA improves model generalization and robustness across domains, paving the way for future domain adaptation research.

## 1. Introduction

In transfer learning, the domain shift problem [1,2,3] occurs when a classifier trained on one data distribution (source domain) performs poorly on another (target domain) data distribution. In image data, this discrepancy may result from disparity in data characteristics between the source and target domains, like variations in image quality, lighting conditions, or even the underlying data-generating processes. Addressing this issue is critical because it directly impacts the generalizability and robustness of machine learning models, particularly in real-world applications where data from multiple distributions are common.

In domain adaptation [4,5,6,7,8], a branch of transfer learning [9,10,11,12], models trained on a source domain are adapted to perform well on a target domain. This solution aims to tackle the problem of domain shift. For several reasons, the source domain often has labeled data, but target domain has less or no labeled data. It is necessary to adjust to unseen, unknown data distributions without using large amounts of labeled data from the target domain. Firstly, it is economical because gathering and annotating large datasets for every new domain is cost- and time-inefficient. Domain adaptation undermines the need for labeled data in the target domain. Secondly, because models are often used in multiple settings, it enhances scalability. Due to effective domain adaptation, consistent model performance is guaranteed in these various settings. Lastly, it addresses domain shift, which improves the model’s generalizability and dependability in a range of situations.

Many approaches have been suggested to address the domain shift problem. They can be broadly classified into the following categories: discrepancy-based methods, adversarial-based methods, reconstruction-based methods, and pseudo-labeling and self-training methods. Minimizing the distribution discrepancy between the source and target domains is the main goal of discrepancy-based techniques like Correlation Alignment (CORAL) [13,14,15] and Maximum Mean Discrepancy (MMD) [16,17,18]. Adversarial-based methods, inspired by Generative Adversarial Networks (GANs) [19,20], use adversarial training to make features from source and target domains indistinguishable, exemplified by Domain-Adversarial Neural Networks (DANNs) [21] and Adversarial Discriminative Domain Adaptation (ADDA) [22,23]. Reconstruction-based methods use autoencoders or other reconstruction mechanisms to learn domain-invariant features by reconstructing input data from both domains [24]. Creating pseudo-labels for target domain data and iteratively improving the model using these labels are the foundations of pseudo-labeling and self-training techniques [25].

Most existing unsupervised domain adaptation (UDA) methods of transfer learning primarily bridge the gap between domains using Maximum Mean Discrepancy (MMD) or Correlation Alignment (CORAL). These techniques focus on aligning first-order and second-order statistics, such as mean and variance, between different distributions while preserving the discriminative power of the labeled source data. Although these methods can reduce domain discrepancy, they do not eliminate it. Consequently, target samples located near the edges of clusters or far away from their respective class centers tend to be misclassified by the decision hyperplanes trained on the source domain. Some works have attempted to minimize empirical conditional distribution divergences by leveraging pseudo-labels to align class-wise means. However, none have explored angular-based distance metrics that guarantee intra-class closeness and inter-class separation within the target domain while also minimizing entropy. Additionally, other approaches utilize convolutional neural networks (CNNs) to extract features from both source and target domains. However, CNNs often fail to preserve the global structure of the data. Despite the potential of these strategies, no existing approach has unified them into a single framework to comprehensively address these challenges. In [26], a transfer learning approach integrates UMAP for dimensionality reduction and Capsule Neural Networks for deep feature extraction. Transfer Adaptive Boosting and Random Forest are used for domain adaptation and classification.

To tackle these issues together, we present a novel domain adaptation framework, DDASLA, that combines angular loss [27], Local Maximum Mean Discrepancy (LMMD) [28], and entropy minimization [29] in a unified manner. This method takes advantage of the strengths of each individual loss function to more effectively address the domain shift issue. In metric learning, angular loss ensures that the angular distance (angle) between samples of the same class (anchor-positive pair) is less than the angle between samples of different classes. This approach encourages the model to learn features that are not only discriminative but also insensitive to rotations and other transformations, resulting in cross-domain consistency. By focusing on angular relationships, the model can distinguish between classes in a more robust manner. Local Maximum Mean Discrepancy (LMMD) is an extension of MMD that considers the data’s local structure. It determines the distribution disparity between the source and target domain distributions within local neighborhoods, allowing for more precise feature alignment. This ensures that comparable samples from various domains are tightly clustered in the feature space. Entropy minimization promotes accurate predictions by reducing the entropy of the output probability distribution for target domain samples, allowing the decision boundary to accommodate more target domain data.

**Figure 1:**
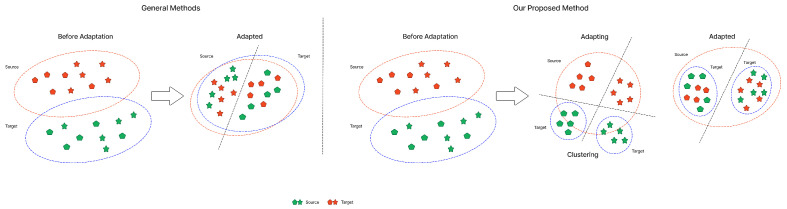
Illustration of domain adaptation, where on the left, class representations (triangles and circles) are initially misaligned due to domain shift. After applying domain adaptation, these classes are aligned, though clustering is suboptimal. The right side demonstrates our method, which integrates angular loss, LMMD, entropy minimization, and an attention mechanism, achieving superior class alignment and clustering, thereby improving accuracy and robustness.

Figure 1 illustrates a general domain adaptation scenario and the proposed framework, DDASLA. The first half initially shows that misalignments occur due to domain shift. Although traditional domain adaptation aligns these classes, it does not achieve optimal clustering as shown in the figure. The second half of the figure shows the effect of our proposed method on the alignment of domains. This approach accomplishes better clustering and more successfully aligns similar classes from both domains, increasing accuracy and robustness across domain boundaries.

The key contributions of this paper are summarized as follows:

- This is, to our knowledge, the first effort to jointly learn discriminative deep features by integrating angular, LMMD, and entropy losses into a unified framework for enhanced feature alignment and decision boundary refinement across domains.
- We incorporate a self-attention mechanism that enhances the model’s focus on the most relevant parts of the data, leading to improved feature learning and domain adaptation.
- Extensive empirical evaluations on multiple benchmark datasets demonstrate that DDASLA consistently outperforms state-of-the-art (SOTA) domain adaptation approaches.

## 2. Related Work

This section reviews various primitive methods and recent advances in the field of domain adaptation, highlighting key contributions and positioning our proposed approach in the context of existing work. Ref. [30] gave a graph neural network-based domain adaptation approach for sensor-based human activity recognition (HAR), enhancing model generalization with limited annotated data. Ref. [31] addressed domain adaptation in activity recognition using binary sensor data from smart environments. It introduced a three-step method: sensor clustering, activity-based temporal alignment, and classifier ensemble mapping. Ref. [32] explored multi-source domain adaptation (MSDA) for smart building applications like activity recognition and occupancy estimation, addressing the challenge of limited labeled data in target domains retrieved by sensors. Ref. [33] presented a sensor-driven domain adaptation method for remote sensing image classification, leveraging sensor invariance to infer labels on unlabeled data in the target domain.

Adversarial methods in deep-domain adaptation have attracted considerable attention because of their effectiveness in learning domain-invariant features through adversarial training. One of the pioneering works in this area is the Domain-Adversarial Neural Network (DANN) proposed by [21]. DANN employs a gradient reversal layer to encourage the feature extractor to produce indistinguishable features between the source and target domains. Building on this approach, Ref. [22] introduced Adversarial Discriminative Domain Adaptation (ADDA), which uses a two-stage training process involving separate encoders for the source and target domains. DIRT-T, proposed by [34], however, undergoes virtual adversarial training and utilizes a teacher model for alignment along with conditional entropy. CDAN, proposed by [35], is another adversarial-based approach that applies domain alignment by integrating features with task knowledge. CoTMix, proposed by [36], is a contrastive domain adaptation method primarily aimed at time-series problems. Cycle-Consistent Adversarial Domain Adaptation (CyCADA), proposed by [37], is another notable contribution in the field of adversarial learning. This method combines adversarial training with cycle-consistency constraints, ensuring that the adapted features are domain-invariant and preserve the semantic content of the input data. These adversarial approaches have shown substantial improvements in various domain adaptation tasks, highlighting their potential to achieve robust and effective domain adaptation.

Researchers have explored various discrepancy-based loss functions integrated into domain adaptation frameworks proposed by [38]. These methods aim to minimize statistical discrepancies between the source and target domains. Maximum Mean Discrepancy (MMD) loss, used by [16], measures the difference in source and target domain distributions in a reproducing kernel Hilbert space. Correlation Alignment (CORAL) loss, as proposed by [13], aligns second-order statistics of features from source and target domains, enhancing feature transferability. These approaches have shown success in various applications, highlighting their efficacy in aligning feature distributions and improving model performance. Ref. [39] introduced a method that integrates joint domain alignment with discriminative feature learning to enhance unsupervised deep-domain adaptation. The method demonstrates significant improvements in performance for tasks such as image classification, highlighting the effectiveness of jointly optimizing domain alignment and feature discrimination in achieving robust domain adaptation. A novel method called Subspace-based Transfer Joint Matching with Laplacian Regularization (STJML) was proposed in [40]. This method jointly aligns subspace representations and re-weights instances across domains while minimizing both marginal and conditional distribution shifts. It also preserves the data’s intrinsic structure through Laplacian regularization. Ref. [41] introduced a kernelized framework for domain adaptation that integrates kernel methods with domain alignment techniques, emphasizing the importance of feature space transformation and distribution alignment. Ref. [42] proposed an unsupervised multi-source visual domain adaptation (MSVDA) method that leverages data from multiple labeled source domains to improve classification in a target domain with unlabeled data. Ref. [43] proposed the Angular-Based Unsupervised Domain Adaptation Framework (AUDAF) that uses pseudo-labels from the k-Nearest Neighbor classifier and formulates an objective function that aligns these angular differences, enhancing domain alignment. Experiments on benchmark datasets showed that AUDAF improves classification accuracy over existing methods.

Proposed by [44], High-order Moment Matching (HoMM) is an advanced method in domain adaptation that extends the concept of traditional moment-matching techniques to higher-order moments. HoMM aims to capture more complex statistical properties of the data distributions by aligning not only the means and variances but also higher-order moments. Ref. [28] employed Local Maximum Mean Discrepancy (LMMD) within their Deep Subdomain Adaptation Network (DSAN) to enhance domain adaptation for image classification. LMMD extends traditional Maximum Mean Discrepancy (MMD) by incorporating local neighborhood information, which allows for a more precise alignment of feature distributions across subdomains. Ref. [45] also proposed a framework for addressing domain shift in image classification. Their approach combines Local Maximum Mean Discrepancy (LMMD) for subdomain alignment, correlation alignment to reduce domain discrepancies, and entropy regularization to enhance robustness in unlabeled target domains. Experiments on benchmark datasets show their methods outperform existing domain adaptation techniques. Ref. [46] addressed the challenge of semisupervised learning under distribution mismatch by proposing a method to assess and quantify dataset similarity before applying semisupervised techniques.

Reconstruction-based approaches focus on learning domain-invariant representations by reconstructing data from both the source and target domains. Ref. [47] introduced Deep Reconstruction–Classification Networks (DRCNs), which simultaneously perform reconstruction and classification tasks. This approach uses autoencoders to learn domain-invariant features by reconstructing data from both domains while preserving discriminative information. Ref. [48] tackles imbalanced domain adaptation by introducing a self-adaptive framework that leverages a deep sparse autoencoder to balance and adapt feature distributions across domains.

Pseudo-labeling and self-training methods are designed to iteratively refine models by generating pseudo-labels for target domain data. One influential approach is the work by [25], which proposes the use of pseudo-labels to enhance semisupervised learning. Another notable method is the self-training approach introduced by [49], which combines pseudo-labeling with consistency regularization. These techniques demonstrate the effectiveness of leveraging target domain data, even with limited labels, to improve model performance and generalization across domains. Ref. [50] proposed a unified framework for visual domain adaptation that utilizes Linear Discriminant Analysis (LDA) enhanced by pseudo-labels. Their approach effectively reduces the domain discrepancy between source and target domains by optimizing the LDA projections using pseudo-labels generated from the target domain, thereby improving classification accuracy in the target domain.

Other approaches include the work of [51] where they developed a continual unsupervised domain adaptation framework specifically designed for data-constrained environments. Similarly, Ref. [52] introduced a source-free domain adaptation method for histopathological images, utilizing domain-centroid-guided progressive teacher-based knowledge distillation. This approach effectively enhances adaptation to target domains in the absence of source data, yielding significant improvements in medical imaging performance.

Triplet Loss is a metric learning technique introduced by [53] that ensures that samples from the same class are closer to each other compared to samples from different classes. Angular loss, introduced by [27], enhances deep metric learning by focusing on angular distances between feature vectors. This method imposes an angular margin between classes, which leads to a more discriminative feature space.

Entropy minimization aims to refine the decision boundaries by reducing the entropy of the output probability distribution for target domain samples. Ref. [29] showed that minimizing entropy helps in enhancing the confidence of predictions, which is beneficial for improving the model’s performance on the target domain by encouraging more decisive outputs.

An attention mechanism is integrated into our framework to focus on relevant parts of the data and suppress less informative features. The work by [54] introduces the idea of self-attention, which enables the model to estimate the importance of different features dynamically. By incorporating attention mechanisms, our method can better capture and emphasize critical features.

Combining these components allows our approach to address domain adaptation from multiple perspectives: angular loss enhances feature learning and separability, LMMD ensures local alignment of features, entropy minimization refines decision boundaries, and the attention mechanism focuses on important features. This comprehensive strategy leads to improved alignment, better generalization, and enhanced performance across domains.

## 3. Methodology

**Figure 2:**
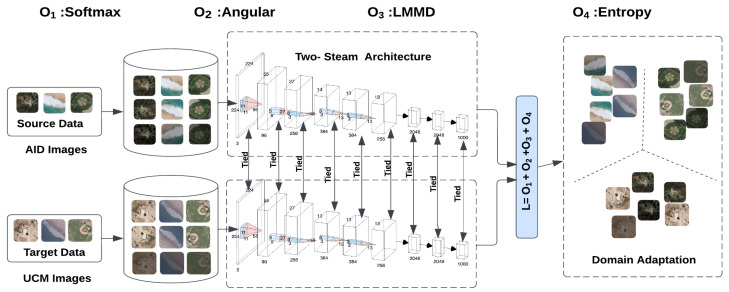
Illustration of the proposed architecture, comprising two identical and weight-sharing modified ResNet18 models. Angular loss and Local Maximum Mean Discrepancy (LMMD) loss are computed from the source and target features, while entropy minimization is applied solely to the target features, facilitating effective domain adaptation and feature alignment.

In this section, we elaborate on the proposed Unified Domain Discrepancy Adaptation Framework, DDASLA, in detail. We utilize a weight-sharing CNN architecture, specifically a ResNet model, with one stream processing the source data and the other handling the target data, as illustrated in Figure 2. Additionally, we incorporate a self-attention mechanism into the architecture. What sets our approach apart is the joint learning of angular loss, domain loss, and entropy loss while preserving the global structure of the data.

In this work, we address the unsupervised domain adaptation (UDA) problem. Let $D_{s}={\left\{x_{s}^{i},y_{s}^{i}\right\}}_{i=1}^{n_{s}}$ represent the source domain with $n_{s}$ labeled data samples, and $D_{t}={\left\{x_{t}^{i}\right\}}_{i=1}^{n_{t}}$ represent the target domain with $n_{t}$ unlabeled data samples, where both $x_{s}$ and $x_{t}$ share the same dimensionality $x_{s},x_{t}\in R^{d}$. The shared parameters to be learned are denoted by Θ. The learned deep features in the bottleneck layer for the source and target streams are represented by $H_{s}\in R^{b\times L}$ and $H_{t}\in R^{b\times L}$, respectively, where *b* is the batch size during training, and *L* is the number of neurons in the bottleneck layer. Detailed descriptions of the architecture, attention mechanism, and loss function are provided below.

### 3.1. ResNet18 Model

ResNet18 is an extensively utilized convolutional neural network architecture known for its deep residual learning framework. It consists of 18 layers with residual connections that help mitigate the vanishing gradient problem, allowing for the training of very deep networks. The basic building block of ResNet18 is the residual block, which includes two convolutional layers and a shortcut connection that adds the input directly to the output. This architecture provides a strong foundation for feature extraction in our domain adaptation tasks.

### 3.2. Attention Mechanism

To enhance the feature extraction capability of ResNet18, we integrate an attention mechanism into its architecture, specifically before the fully connected layer. The attention mechanism enables the model to concentrate on the most significant parts of the input, thereby improving its ability to capture crucial features for domain adaptation.

#### 3.2.1. Self-Attention Mechanism

The self-attention mechanism, also known as scaled dot-product attention, computes a weighted sum of input features, with weights dynamically determined based on the association between different parts of the input. This mechanism selectively emphasizes significant features while suppressing less relevant ones, thus enhancing feature extraction.

Given an input feature map $H\in R^{b\times L}$, the self-attention mechanism is described by the following steps:

**1. Linear Projections:** The input feature map *H* is linearly projected into three different spaces—queries *Q*, keys *K*, and values *V*—using learned projection matrices $W_{Q}$, $W_{K}$, and $W_{V}$, respectively:

(1) $$Q=HW_{Q},\;K=HW_{K},\;V=HW_{V},$$

where $Q,K,V\in R^{b\times L_{k}}$.

**2. Scaled Dot-Product Attention:** The attention scores are obtained by calculating the dot product of the query and key matrices, followed by scaling by $\frac{1}{\sqrt{L_{k}}}$ to stabilize the gradients:

(2) $$A=\mathrm{softmax}\left(\frac{QK^{T}}{\sqrt{L_{k}}}\right)$$

where $A\in R^{b\times b}$ represents the attention weights.

**3. Weighted Sum:** The output of the self-attention mechanism is obtained by matrix multiplication of the attention weight matrix *A* and the value matrix *V*:

(3) O=AV

where $O\in R^{b\times L_{k}}$ is the attention output.

**4. Linear Projection:** Finally, the attention output *O* is projected back to the original feature space using a learned projection matrix $W_{O}$:

(4) $$Z=OW_{O}$$

where $Z\in R^{b\times L}$ is the final output of the attention mechanism.

#### 3.2.2. Integration into ResNet18

In our architecture, the attention mechanism is integrated before the fully connected layer of ResNet18. The feature map H output from the last convolutional layer of ResNet18 is passed through the attention mechanism to produce a refined feature map *Z*, which is then fed into the fully connected layer for classification.

#### 3.2.3. Advantages for Domain Adaptation

The incorporation of the attention mechanism before the fully connected layer confers several advantages for domain adaptation. Firstly, the attention mechanism enhances feature discriminability by helping the model concentrate on the most important parts of the input, resulting in more discriminative features that are better suited for distinguishing between different classes, even in the presence of domain shifts. Secondly, it improves generalization by emphasizing important features and suppressing irrelevant ones, thereby enhancing the model’s ability to generalize to the target domain and improving overall performance in domain adaptation tasks. Thirdly, the dynamic weighting of features by the attention mechanism enables the model to adapt more effectively to variations in the input data, making it more robust to domain shifts. Overall, the integration of the attention mechanism into the ResNet18 architecture before the fully connected layer significantly enhances the model’s capability for domain adaptation by improving feature discriminability, generalization, and robustness to domain shifts.

### 3.3. Loss Functions

As per the theory laid down by [55], the source domain loss and the domain discrepancy loss should be incorporated in a domain adaptation method, i.e.,

(5) $$L\left(\theta |X_{s},Y_{s},X_{t}\right)=L_{s}+L_{d}$$

(6) $$L_{s}=\frac{1}{n_{s}}\sum_{i=1}^{n_{s}}J\left(h_{s}^{i},y_{s}^{i}\right)$$

where $L_{s}$, $L_{d}$, and J(.,.) denote the classification loss in the source domain, the domain discrepancy loss, and the cross-entropy loss function, respectively. The domain discrepancy loss consists of the following loss functions.

**Figure 3:**
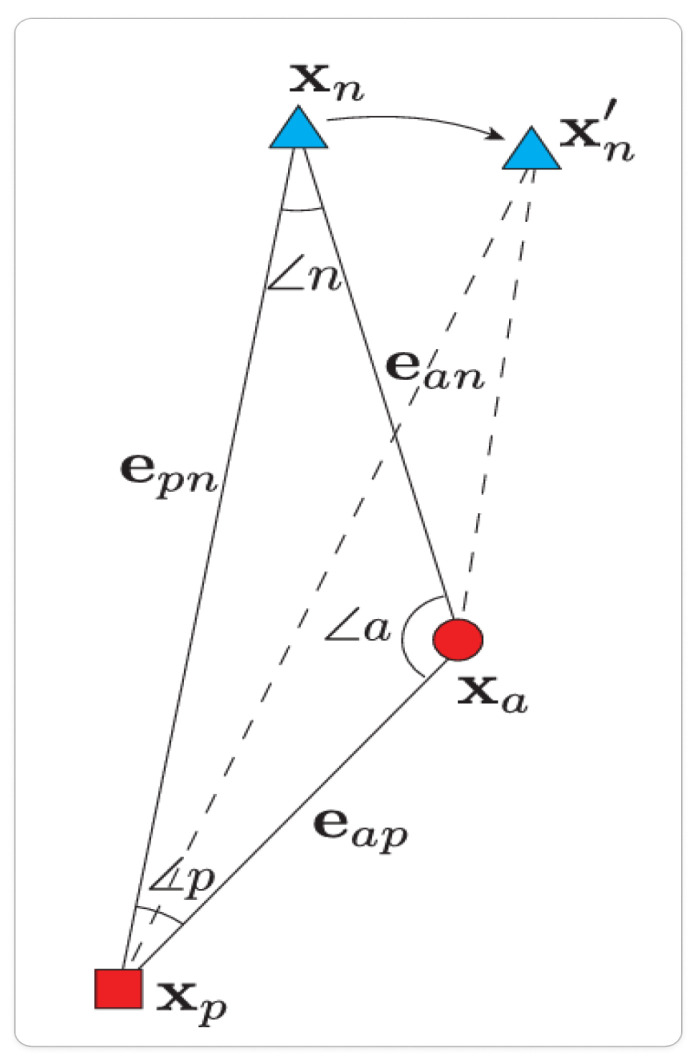
Illustration of angular loss, depicting the angle between anchor-positive and anchor-negative vectors (∠n). This visualization highlights the aim to maximize separation between dissimilar points while minimizing the angle with similar points [27].

**Angular loss** [27] improves feature separability by focusing on angular distances between feature vectors as shown in Figure 3. It enforces an angular margin between classes, leading to a more compact and discriminative feature space.

(7) $$L_{a}=\sum_{i}{\left[∥h_{i}^{a}-h_{i}^{p}{∥}_{2}^{2}-4\tan^{2}\left(\alpha \right){∥h_{i}^{a}-h_{i}^{n}∥}_{2}^{2}\right]}_{+}$$

where $h_{i}^{a}$, $h_{i}^{p}$, and $h_{i}^{n}$ denote the anchor, positive, and negative samples, respectively, and α denotes the constraining angle. Angular loss provides a more robust metric for feature discrimination by considering the angular relationships between samples, which can be particularly beneficial when dealing with complex domain shifts.

**Local Maximum Mean Discrepancy (LMMD)** [28] is employed to align feature distributions at a more granular level by incorporating local neighborhood information. LMMD guarantees that similar samples from different domains are mapped closer in the feature space, and it is defined as follows:

(8) $$L_{l}=\sum_{i,j}{∥E_{h_{i}∼D_{s}}\left[h_{i}\right]-E_{h_{j}∼D_{t}}\left[h_{j}\right]∥}^{2}$$

By aligning the local feature distributions between the source and target domains, LMMD helps reduce the domain discrepancy, thereby facilitating more effective domain adaptation.

**Entropy Minimization** [29] encourages the model to make confident predictions by reducing the entropy of the output probability distribution for target domain samples. The entropy loss is defined as follows:

(9) $$L_{e}=-\sum_{i}\sum_{c}p\left(y_{c}|h_{i}\right)\log p\left(y_{c}|h_{i}\right)$$

where $p(y_{c}|h_{i})$ is the predicted probability of class *c* for sample $h_{i}$. By minimizing the entropy, the model is driven to make more confident and decisive predictions for target domain samples, which can improve the overall classification performance in the target domain.

### 3.4. Overall Loss Function

The final loss function combines the above losses along with the classification loss to form a comprehensive objective for domain adaptation:

(10) $$L=L_{s}+\lambda_{1}L_{a}+\lambda_{2}L_{l}+\lambda_{3}L_{e}$$

where $\lambda_{1}$, $\lambda_{2}$, and $\lambda_{3}$ are hyperparameters that balance the contributions of the angular, LMMD, and entropy losses, respectively. By integrating these loss functions and the attention mechanism into the ResNet18 architecture, DDASLA aims to enhance feature discriminability, align local data distributions, refine decision boundaries, and focus on relevant features, thereby improving domain adaptation performance.

## 4. Experiments and Results

In this section, we discuss the datasets used for our experiments, describe the implementation details, present the results of domain adaptation approaches, and compare them with our proposed method, DDASLA.

**Figure 4:**
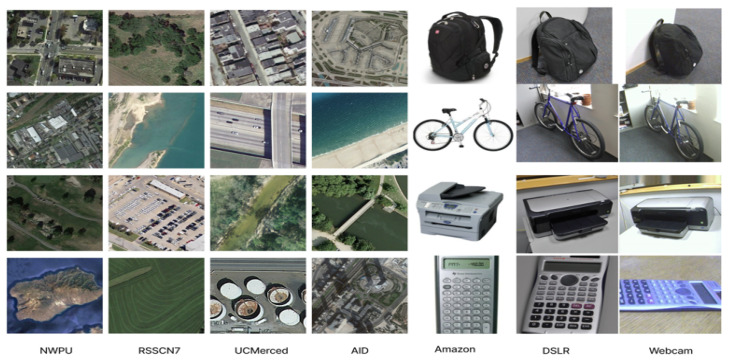
A few samples of the remote sensing dataset (first four columns consisting of the NWPU-RESISC45, RSSCN7, UCMerced, and AID datasets) and the Office31 dataset (last three columns from the right consisting of Amazon, Webcam, and DSLR).

We conducted our experiments on two datasets: the Office dataset and the remote sensing dataset. Each dataset poses unique challenges and provides a comprehensive evaluation of our domain adaptation method. A few samples of the remote sensing dataset and Office31 dataset are shown in Figure 4.

### 4.1. Office Dataset

The Office dataset is a widely used benchmark dataset for domain adaptation tasks. It consists of images collected from three distinct domains: Amazon (A), DSLR (D), and Webcam (W). These domains represent different environments with varying image quality and backgrounds, making them ideal for evaluating domain adaptation techniques. Each domain contains images from 31 categories, including common office items such as keyboards, monitors, and mice. For our experiments, we performed several domain adaptation tasks by selecting different combinations of source and target domains, including A → D, D → W, and W → A.

### 4.2. Remote Sensing Datasets

We also evaluated our method on remote sensing datasets, specifically focusing on the UCMerced Land Use Dataset, AID, NWPURESISC45, and RSSCN7. These datasets present a different set of challenges due to variations in resolution, geographical locations, and seasonal changes.

- **UCMerced Land Use Dataset (UC Merced):** The UC Merced dataset includes 21 land-use classes each containing 100 images, captured at a 30 cm resolution.

- **AID:** The AID dataset comprises aerial images from 30 different land-use categories, with a varying number of images per category, ranging from 220 to 420.

For our experiments, we selected nine common classes between UC Merced and AID, such as agricultural, residential, and industrial areas, to form a combined dataset for domain adaptation tasks.

- **NWPURESISC45:** This dataset includes 45 scene classes with 700 images per class, captured from various remote sensing platforms.

- **RSSCN7:** The RSSCN7 dataset contains images from seven scene categories, with 400 images per category, taken under different weather conditions and seasons. The AID dataset has 30 different scene classes and about 200 to 400 samples of size 600 × 600 in each class. The UCM dataset has 21 classes of land-use images. There are 100 images (256 × 256 pixels) for each class. In our research, we only used the common classes—baseball, beach, dense residential, forest, medium residential, parking, river, sparse residential, and strong tanks—in the AID and UCM datasets. In the same manner, the NWPU-RESISC45 dataset has 45 scene classes, and the RSSCN7 dataset has seven classes: grassland, farmland, industrial and commercial regions, river and lake, forest field, residential region, and parking lot. We have chosen six common classes—grassland, industrial, river, forest, residential, and parking lot—for our research.

### 4.3. Implementation Details

As explained earlier, for implementing DDASLA, we used a ResNet18 backbone with integrated attention mechanisms, optimized with the aforementioned loss functions: angular loss, LMMD, and entropy minimization. Our experiments were conducted using the following configurations: a batch size of 16, 20 training epochs, an initial learning rate of $10^{-2}$, an L2 weight decay of $5\times 10^{-4}$, and a momentum of 0.9. To manage the learning rate effectively, we applied a step decay, with an initial learning rate of 0.01, which decayed by 0.1 every 10 epochs. The optimizer used was Stochastic Gradient Descent (SGD) with the updated learning rate divided by 10, incorporating the specified momentum and weight decay parameters. The hyperparameters $\lambda_{1}$, $\lambda_{2}$, and $\lambda_{3}$ and angular-margin value were carefully tuned using Optuna Optimization to balance the contributions of the different loss components.

### 4.4. Comparison with Other Methods

To evaluate the effectiveness of DDASLA, we compared it with several classical and deep-domain adaptation methods:

**DDC (Deep-Domain Confusion)** [16]: This method employs Maximum Mean Discrepancy (MMD) to align the distributions of source and target domains. The MMD loss helps in reducing the domain gap by matching the feature distributions across domains.

**CORAL (Correlation Alignment)** [13]: CORAL aligns the second-order statistics of source and target domain distributions by minimizing the difference in their covariance matrices. This method enhances the generalization ability of the model to the target domain.

**JDDA (Joint Distribution Adaptation)** [39]: JDDA enhances domain adaptation performance by jointly aligning the marginal and conditional distributions of the source and target domains. It combines MMD-based alignment with a classifier adaptation strategy.

**HoMM (Higher-order Moment Matching)** [44]: HoMM extends the idea of moment matching by aligning higher-order moments (beyond the second order) of the source and target distributions, thus capturing more complex domain shifts.

**SACAEM (Subdomain Adaptation via Correlation Alignment with Entropy Minimization)** [45]: SACAEM improves unsupervised domain adaptation by combining Local Maximum Mean Discrepancy for local feature alignment, correlation alignment to match feature correlations between domains, and entropy minimization to promote confident predictions on the target domain.

### 4.5. Results and Analysis

Table 1 presents a performance comparison of five consecutive runs of various methods across different datasets and domain adaptation scenarios. For both datasets, UCM→AID and AID→UCM, our proposed framework, DDASLA, outperforms all other deep-domain adaptation methods, showing improvements of 9.3% and 8.4% over the baseline, respectively. This significant gain signifies the effectiveness of our approach in bridging the distribution gap between domains. The second-best performance by SACAEM, compared to the poor results from the DCC method, suggests that both datasets are locally distributed, necessitating the use of LMMD and entropy loss to further reduce the distribution gap. However, a discriminative loss remains essential to prevent misclassifying the target domain data samples located near the cluster edges or distant from their respective class centers.

Nevertheless, a discriminative loss is crucial to avoid misclassifying target domain samples that are located near cluster boundaries or distant from their corresponding class centers.

Similar trends are observed in the NWPU→RSSCN7 dataset. Notably, the higher-order moment-matching method, HoMM4, outperforms all other methods, indicating that matching higher-order moments significantly reduces the distribution gap between domains. While DDASLA trails HoMM4 by 1%, it still outperforms all other approaches, underscoring the importance of each loss function integrated into our framework.

On the Office31 dataset, DDASLA consistently outperforms all other approaches across all tasks. SACAEM ranks second in most tasks, except for the A→D and W→D tasks. This result highlights the effectiveness of minimizing entropy loss and applying LMMD in achieving robust domain-invariant features. The second-place performance of Gaussian MMD in the A→D task suggests that the data is not linearly separable, and minimizing the first-order distribution in kernel space yields better accuracy. However, it still falls short of DDASLA’s accuracy due to the inclusion of other critical objectives in it. The second-place ranking of higher-order moment matching in the W→D task further demonstrates that matching higher-order moments can enhance model performance.

**Figure 5:**
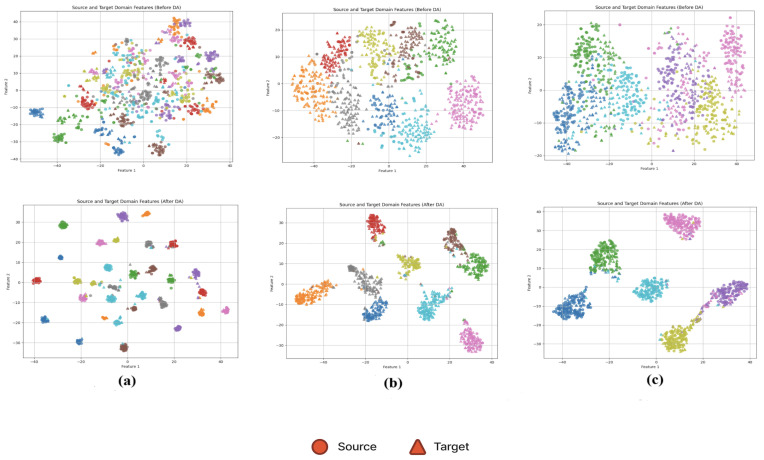
Feature representation of data before and after applying domain adaptation (DA) with our proposed method, DDASLA, across different datasets. Circle markers represent source domain samples, and triangle markers represent target domain samples. (**a**) Amazon → Webcam, (**b**) UCMerced → AID, and (**c**) NWPU → RSSCN7.

**Figure 6:**
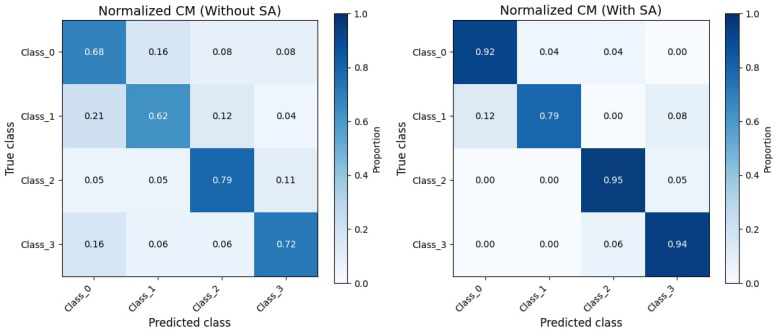
Confusion matrix illustrating proposed model performance with and without self-attention.

Overall, DDASLA significantly improves the feature representation in the target domain as shown in Figure 5. By simultaneously optimizing for angular loss, LMMD, and entropy minimization, and incorporating an attention mechanism, we achieve a more robust and generalized model that can effectively bridge the domain gap between the Amazon and Webcam datasets. The confusion matrix shown in Figure 6 indicates that self-attention provides valuable context that improves the model’s ability to achieve clearer class separation and a reduction in classification errors.

### 4.6. Performance Comparison and Analysis

**Figure 7:**
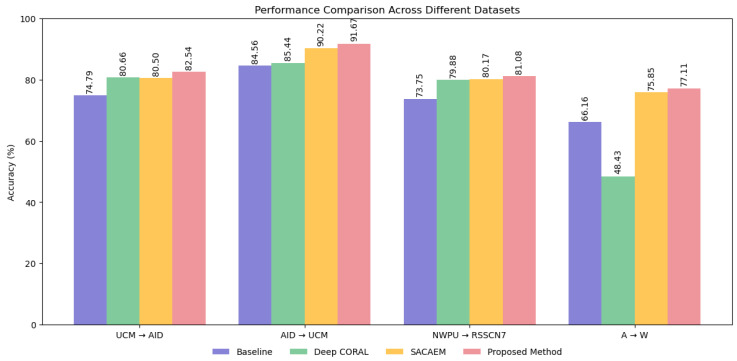
Performance comparison of Baseline, Deep CORAL, SACAEM, and our proposed method, DDASLA, across four domain adaptation datasets.

Figure 7 presents a comparative analysis of DDASLA against the baseline, Deep CORAL [13], and SACAEM [45] across various domain adaptation tasks. Our method consistently outperforms other approaches across all dataset combinations. For the UCM → AID task, we achieve 82.54% accuracy, surpassing the baseline by 7.75% and SACAEM by 2.04%. Similarly, in the AID → UCM task, our method reaches 91.67% accuracy, demonstrating a 7.11 and 1.45% improvement over the baseline and SACAEM, respectively. In the challenging NWPU → RSSCN7 scenario, our method achieves 81.08% accuracy, showcasing its ability to handle complex domain shifts in remote sensing applications. For the Office31 dataset (A → W task), we attain 77.11% accuracy, marking a substantial improvement of 10.95 and 1.26 percentage over the baseline and SACAEM, respectively. The superior performance of our method can be attributed to the synergistic effect of angular loss, Local Maximum Mean Discrepancy (LMMD), entropy minimization, and the attention mechanism. These components collectively enhance feature discrimination, local distribution alignment, and confident predictions on the target domain. These results consistently demonstrate our method’s effectiveness in bridging domain gaps across diverse datasets, underlining its potential as a robust solution for unsupervised domain adaptation challenges.

### 4.7. Parameter Sensitivity

**Figure 8:**
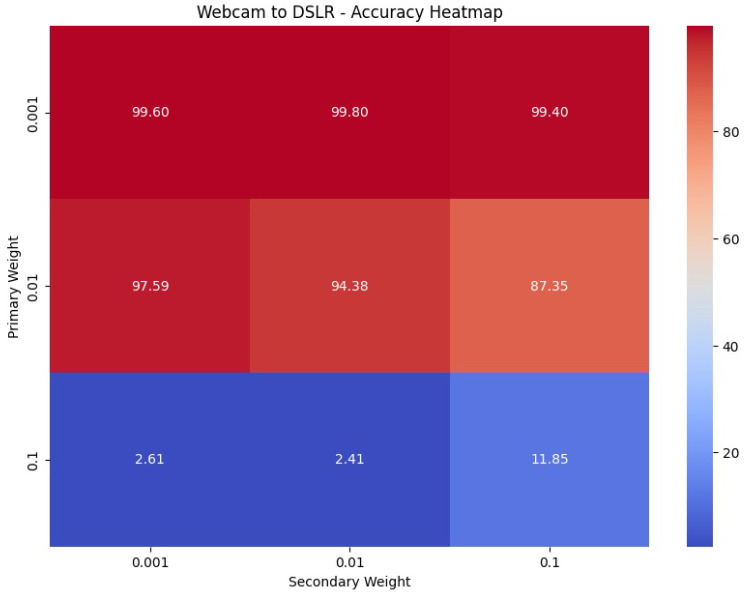
Parameter sensitivity analysis for different combinations of weights for angular loss ($\lambda_{1}$) and LMMD loss ($\lambda_{2}$) in the Webcam to DSLR dataset adaptation.

The parameter sensitivity analysis depicted in Figure 8 evaluates the performance of DDASLA under various combinations of weights for angular loss ($\lambda_{1}$) and LMMD loss ($\lambda_{2}$). It was observed that the combination of $\lambda_{1}=0.1$ and $\lambda_{2}=0.001$ yielded the best performance, indicating that a moderate emphasis on angular loss and a smaller emphasis on LMMD loss effectively balances the network’s ability to learn discriminative features and align the distributions across domains. This combination provided the most robust results in the domain adaptation from Webcam to Amazon, optimizing both feature discrimination and domain alignment. Similar experiments were conducted for other domain adaptation tasks, and the weights were adjusted accordingly to achieve optimal performance across all scenarios.

### 4.8. Cost Analysis

We implemented our experiments using the PyTorch (2.2.2+cu118) framework, leveraging pretrained models from the PyTorch library to ensure robust feature extraction and efficient training. All experiments were conducted on a system equipped with an Nvidia RTX A4000 GPU with 16GB RAM, providing the necessary computational power and memory for handling the complex models and large datasets involved in our work. Around 13 M parameters, 3.2 GFLOPS with inference time of 4–6 images/ms is observed in our experiments with 20 training epochs. The integration of multiple loss functions and self-attention mechanisms increases the model’s computational cost, impacting both training time and memory usage. To evaluate the practicality of our approach, we compared it with a baseline model that uses only cross-entropy loss without additional losses or self-attention mechanisms. Our results show that the proposed model increases computational demands by approximately 20%, achieving significant gains in domain adaptation accuracy and demonstrating a favorable trade-off between cost and performance. To manage these computational demands, we adopted a lightweight self-attention configuration and carefully optimized loss weightings.

### 4.9. Ablation Study

We conducted an ablation study to determine whether the loss functions included in the proposed framework are redundant. In this study, we removed one objective at a time, performed experiments, and observed the impact on the model’s performance. This allowed us to determine the contribution of each loss function. The results of this study are presented in Table 2, which reveals that removing any of the objectives leads to a decrease in the model’s performance of DDASLA across all datasets. This analysis demonstrates that each loss function plays a crucial role in achieving the optimal performance of the model. Simultaneously, we checked the effect of the attention mechanism on the model’s performance. We observed an increase in performance due to the attention mechanism, as shown in Table 2.

## 5. Conclusions and Future Work

In this work, we developed a unified deep-domain adaptation framework, DDASLA, by integrating angular loss, Local Maximum Mean Discrepancy (LMMD), entropy minimization, and an attention mechanism within a CNN model to achieve domain-invariant feature extraction. DDASLA effectively reduces domain shift and enhances feature separability, aligns local data distributions, and refines decision boundaries. The inclusion of the attention mechanism significantly improved feature extraction and generalization across domains. Extensive experiments on the Office31 and remote sensing datasets demonstrated the superior performance of our method compared to existing techniques like MMD (DDC), Deep CORAL, JDDA, and SACAEM. These results suggest that our framework provides a robust solution for domain adaptation challenges, making it highly suitable for applications requiring strong generalization across varying data distributions. The work can be further extended by focusing on extending the approach to adding advanced architectures and more loss functions and optimizing them to address broader domain adaptation scenarios.

## Figures and Tables

**Table 1:** Performance comparison across various datasets and methods. In the context of the Office31 dataset, “A” refers to Amazon, “W” to Webcam, and “D” to DSLR.

Methods	UCM → AID	AID → UCM	NWPU → RSSCN7	A → W	W → A	D → A	A → D	W → D	D → W
Baseline	74.89 ± 0.2%	84.56 ± 0.3%	73.59 ± 0.2%	66.16 ± 0.1%	50.83 ± 0.3%	51.72 ± 0.3%	67.51 ± 0.2%	99.00 ± 0.1%	94.89 ± 0.3%
MMD (DDC) [16]	74.01 ± 0.3%	80.11 ± 0.2%	78.12 ± 0.3%	65.31 ± 0.1%	51.15 ± 0.2%	51.59 ± 0.2%	69.08 ± 0.3%	97.5 ± 0.3%	95.37 ± 0.1%
Gaussian MMD [16]	74.81 ± 0.3%	81.41 ± 0.2%	73.51 ± 0.1%	65.76 ± 0.2%	52.59 ± 0.1%	51.13 ± 0.2%	69.89 ± 0.3%	98.29 ± 0.3%	95.97 ± 0.3%
Polynomial MMD [16]	78.57 ± 0.1%	75.50 ± 0.2%	66.63 ± 0.1%	65.59 ± 0.4%	53.81 ± 0.3%	53.21 ± 0.3%	69.5 ± 0.2%	98.58 ± 0.2%	36.25 ± 0.4%
Deep Coral [13]	79.8 ± 0.3%	85.44 ± 0.3%	79.88 ± 0.2%	48.43 ± 0.4%	50.72 ± 0.2%	47.38 ± 0.3%	31.93 ± 0.3%	54.62 ± 0.2%	86.92 ± 0.2%
JDDA [39]	75.62 ± 0.3%	72.80 ± 0.2%	70.56 ± 0.2%	57.37 ± 0.3%	51.50 ± 0.3%	49.50 ± 0.2%	58.94 ± 0.3%	99.58 ± 0.3%	95.68 ± 0.2%
Homm3 [44]	69.96 ± 0.1%	87.01 ± 0.2%	77.84 ± 0.3%	64.38 ± 0.3%	51.15 ± 0.2%	53.26 ± 0.2%	63.52 ± 0.3%	99.01 ± 0.2%	95.10 ± 0.2%
Homm4 [44]	78.63 ± 0.2%	86.33 ± 0.3%	**81.92 ± 0.3%**	62.89 ± 0.3%	52.68 ± 0.3%	54.49 ± 0.3%	64.06 ± 0.2%	99.40 ± 0.3%	95.60 ± 0.3%
SACAEM [45]	80.32 ± 0.2%	89.82 ± 0.3%	80.01 ± 0.2%	75.6 ± 0.2%	54.30 ± 0.2%	54.35 ± 0.3%	56.83 ± 0.2%	96.09 ± 0.2%	96.13 ± 0.3%
Proposed Method (DDASLA)	**82.4 ± 0.2%**	**91.71 ± 0.1%**	81.16 ± 0.2%	**76.8 ± 0.3%**	**55.46 ± 0.3%**	**56.15 ± 0.2%**	**73.58 ± 0.2%**	**99.70 ± 0.1%**	**96.5 ± 0.2%**

**Note:** Bold values indicate the highest accuracy in each comparison group.

**Table 2:** Ablation study results. In the context of the Office31 dataset, “A” refers to Amazon, “W” to Webcam, and “D” to DSLR.

Ablation	AID → UCM	NWPU → RSSCN7	A → W	W → D	D → W
Without Entropy	90.56%	79.79%	77.11%	99.60%	96.35%
Without LMMD	87.33%	76.17%	70.31%	99.20%	96.23%
Without Angular	91.55%	79.50%	75.47%	99.60%	96.48%
Without Attention	91.22%	81.00%	75.85%	99.00%	95.97%
With Attention	91.67%	81.08%	77.11%	99.80%	96.60%
Proposed Method (DDASLA)	91.67%	81.08%	77.11%	99.80%	96.60%

## Data Availability

The research data supporting this publication are available from the corresponding author upon reasonable request.
